# Editorial: The urogenital microbiota in urinary tract diseases

**DOI:** 10.3389/fcimb.2022.1096168

**Published:** 2022-12-08

**Authors:** Nicole M. Gilbert, A. Lenora Ackerman, Amanda L. Lewis

**Affiliations:** ^1^ Department of Pediatrics, Division of Infectious Diseases, Washington University School of Medicine, St. Louis, MO, United States; ^2^ Department of Urology, David Geffen School of Medicine at UCLA, Los Angeles, CA, United States; ^3^ Department of Obstetrics, Gynecology, and Reproductive Sciences, University of California, San Diego, La Jolla, CA, United States

**Keywords:** bladder, urobiome, vagina, vaginal microbiome, lower urinary track symptoms, *Gardnerella*, *Lactobacillus*, Uropathogenic E coli (UPEC)

The urogenital microbiota (urinary, periurethral, vaginal and penile) has the potential to modulate susceptibility or severity of infections caused by recognized uropathogens (e.g. UTI, urethritis) or other urologic conditions not previously regarded as having a microbial origin (e.g. IC/BPS, interstitial cystitis, bladder cancer). In this Research Topic, a collection of studies used cutting-edge tools to analyze, or re-analyze, urogenital microbiome data from a variety of patient groups to examine associations with urinary tract disorders or between urogenital niches. Other studies developed new methods for analyzing urobiome data or for examining the effect of urogenital bacteria on the mucosa. Finally, a group of studies developed new *in vitro* and *in vivo* model systems to directly examine how specific members of the urogenital microbiome affect recognized uropathogens.

The bioinformatic tools and pipelines used to generate and analyze microbiome data are constantly advancing. New insights can be generated from re-analysis of existing samples or datasets. Joyce et al. re-analyzed the urobiome in >1,000 adult women with a wide range of urinary tract syndromes: asymptomatic, urgency urinary incontinence (UUI), stress urinary incontinence (SUI), urinary tract infection (UTI) and interstitial cystitis/painful bladder syndrome (IC/PBS). Siddiqui et al. re-analyzed 16S rRNA gene sequencing data from >200 individuals with or without mixed urinary incontinence (MUI). The new analytical approaches used in these studies revealed previously unrecognized features of the urobiome. Another consideration in microbiome research is the choice of which 16S hypervariable region to analyze. Heidrich et al. report that the V1V2 regions best capture the taxa present in male urine samples. Together, these studies highlight the importance of revisiting microbiome data as computational tools progress and of empirically determining the best experimental approach for analyzing the urobiome of specific populations, which may be different that what it optimal for other human biological samples such as stool.

A caveat to standard 16S sequence-based microbiome data is that it cannot distinguish live bacteria from dead. Bacterial viability can be demonstrated using extended quantitative urine culture (EQUC), but this method adds a cost and labor burden and does not capture all urobiome species. The DNA-binding dye propidium monoazide (PMA), which can penetrate dead/dying cells and thus prevent PCR amplification, has been used in other microbiome studies to distinguish viable from non-viable bacteria. Lee et al. developed methods for utilizing the PMA-binding PCR assay in urine. With further refinement, PMA-based urine PCR has the potential clinical advantages of more rapid UTI diagnosis and broader organism detection and could identify situations where recalcitrant symptoms following UTI treatment are due to persistence of viable but nonculturable bacteria.

Most of the bacterial genera reported in urobiome studies had previously been recognized as vaginal bacteria. This observation, coupled with the proximity of the urethra to the vaginal introitus, suggests that microbial overlap exists between the urinary tract and the vagina in individual women. Nardos et al. performed microbial network analysis on paired vaginal and catheterized urine samples from women with and without UUI. Echoing previous studies, there was substantial overlap between vaginal and urine samples across all participants. However, the number of shared bacterial genera between the two niches was higher in UUI patients and the most abundantly shared was *Gardnerella*, while controls had lower numbers of shared bacterial genera and the most abundantly shared was *Lactobacillus*. These data suggest that women with UUI could have more frequent bladder exposures to vaginal bacteria or that they have a bladder environment more permissive to persistence of certain vaginal bacteria. Longitudinal studies in women and direct experimental investigations (like those described below) are needed to distinguish these possibilities.

The next step after discovering the existence of viable bacteria in urine samples is determining what the bacteria are doing in the bladder. Answering this question requires new *in vitro* and *in vivo* model systems. Nguyen et al. developed an *in vitro* assay to examine a biologically relevant effect that urogenital bacteria could be having on the bladder mucosa: degradation of the protective glycosaminoglycan GAG layer. This rapid, inexpensive and quantitative assay demonstrated GAG degradation by the uropathogen *Proteus mirabilis* but not by uropathogenic *Escherichia coli* (UPEC) or any of several species of urinary lactobacilli. Johnson et al. examined the ability of urinary *Lactobacillus* isolates to inhibit Gram-negative and Gram-positive uropathogenic model strains and clinical and multi-drug resistant isolates *in vitro.* There was substantial variety in the ability and mode of inhibition among *Lactobacillus* species and strains.

Previous studies developed the first *in vivo* models examining the effect of the frequent and abundant urogenital microbiome member *Gardnerella* on the bladder in mice. *Gardnerella* is cleared from the mouse bladder within 12 hours after transurethral inoculation, but nonetheless caused urothelial exfoliation and triggered recurrent (r)UTI from quiescent intracellular reservoirs (QIRs) of UPEC. In two follow-up studies using this model (O’Brien et al. and Gilbert et al.), RNA-seq analysis revealed that bladders exposed to *Gardnerella* displayed a transcriptional signature of inflammation and urothelial turnover, both in naive mice and those harboring UPEC QIRs. The orphan nuclear receptor Nur77 was induced by *Gardnerella* in mice with QIRs, and additional studies with knockout mice demonstrated that it is required for *Gardnerella* exposure to induce UPEC rUTI (O’Brien et al.). These results established the utility of an RNA-seq approach to identify genes that mediate the effect of urogenital bacteria on UTI susceptibility. In naive mice, bladder exposure to *Gardnerella* paved the way for a subsequent UTI, lowering the dose of UPEC necessary to result in persistent infection (Gilbert et al.).

The articles in this Research Topic highlight the importance of a multi-pronged approach for urogenital microbiome research (**Figure 1**). The field will continue to thrive as it applies cutting-edge technologies and analytical tools to urobiome analysis in order to reveal associations between certain organisms and particular clinical phenotypes, and then uses that information to develop novel experimental model systems to directly address the mechanistic nature of these relationships. In turn, observations made in these model systems will inform future, more targeted clinical investigations of links between the urogenital microbiome and urinary tract diseases. This iterative approach has the greatest promise to yield actionable steps to improve bladder health.

**Figure 1:**
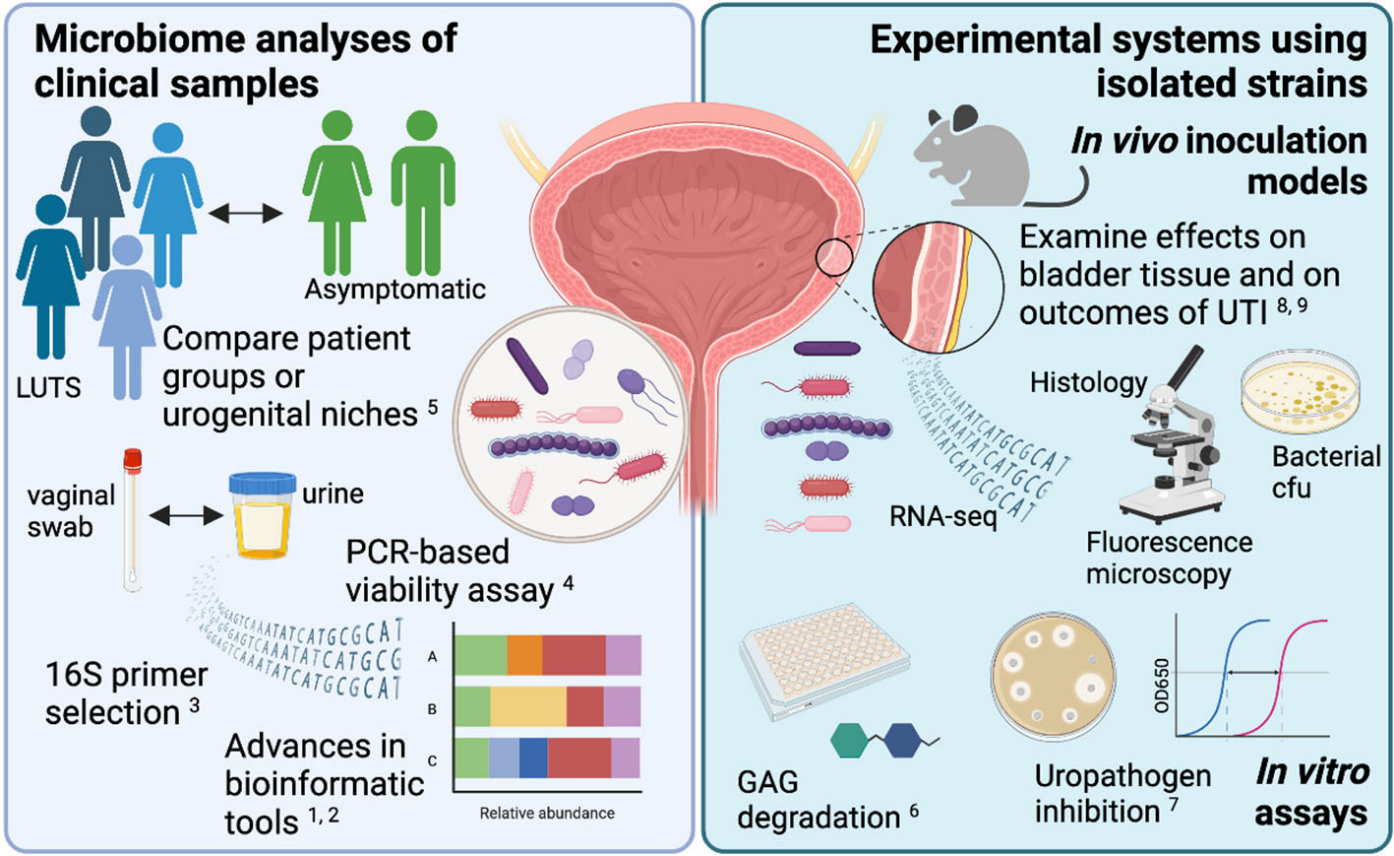
New bioinformatic tools and experimental model systems are refining our understanding of how the urogenital microbiota contributes to urinary tract diseases. The figure was created using BioRender.com.

## Author contributions

NG wrote the initial manuscript draft and all authors edited and approved of the final editorial. NG created the figure. All authors contributed to the article and approved the submitted version.

## Funding

This work was supported by the National Institutes of Health NIAID [R21 AI152049 to AL and NG].

## Conflict of interest

ALA is a consultant for Abbvie, Inc. and Watershed Medical and receives grant support from Medtronic, Inc. and MicrogenDx.

The remaining author declares that the research was conducted in the absence of any commercial or financial relationships that could be construed as a potential conflict of interest.

## Publisher’s note

All claims expressed in this article are solely those of the authors and do not necessarily represent those of their affiliated organizations, or those of the publisher, the editors and the reviewers. Any product that may be evaluated in this article, or claim that may be made by its manufacturer, is not guaranteed or endorsed by the publisher.

